# Supplementary material for: Modeling glioblastoma heterogeneity as a dynamic network of cell states
Source: Mol Syst Biol. 2021 Sep 16;17(9):e10105. doi: 10.15252/msb.202010105 (PMC8444284; doi:10.15252/msb.202010105)
Supplement: Supplementary file 5 — Source Data for Figure 3 [file MSB-17-e10105-s001.zip › Figure3A_sourcedata/GSEA_3065/hallmarks_state4.GseaPreranked.1623416296068/HALLMARK_MTORC1_SIGNALING.html]

Details for gene set HALLMARK\_MTORC1\_SIGNALING[GSEA]

|  || Dataset | state4 |
| Phenotype | NoPhenotypeAvailable |
| Upregulated in class | na\_neg |
| GeneSet | HALLMARK\_MTORC1\_SIGNALING |
| Enrichment Score (ES) | -0.56123006 |
| Normalized Enrichment Score (NES) | -2.0852697 |
| Nominal p-value | 0.0 |
| FDR q-value | 0.0 |
| FWER p-Value | 0.0 |
Table: GSEA Results Summary

  

Fig 1: Enrichment plot: HALLMARK\_MTORC1\_SIGNALING      
 Profile of the Running ES Score & Positions of GeneSet Members on the Rank Ordered List

  

| PROBE | GENE SYMBOL | GENE\_TITLE | RANK IN GENE LIST | RANK METRIC SCORE | RUNNING ES | CORE ENRICHMENT || 1 | MLLT11 |  |  | 267 | 0.182 | -0.0331 | No |
| 2 | CDKN1A |  |  | 319 | 0.171 | -0.0316 | No |
| 3 | SQSTM1 |  |  | 390 | 0.155 | -0.0341 | No |
| 4 | PRDX1 |  |  | 416 | 0.149 | -0.0296 | No |
| 5 | BHLHE40 |  |  | 451 | 0.143 | -0.0269 | No |
| 6 | DDIT4 |  |  | 493 | 0.138 | -0.0257 | No |
| 7 | GAPDH |  |  | 494 | 0.138 | -0.0177 | No |
| 8 | SKAP2 |  |  | 552 | 0.131 | -0.0195 | No |
| 9 | RAB1A |  |  | 671 | 0.118 | -0.0320 | No |
| 10 | BTG2 |  |  | 689 | 0.116 | -0.0281 | No |
| 11 | GSK3B |  |  | 788 | 0.105 | -0.0380 | No |
| 12 | IGFBP5 |  |  | 901 | 0.096 | -0.0508 | No |
| 13 | RIT1 |  |  | 1021 | 0.086 | -0.0653 | No |
| 14 | NMT1 |  |  | 1049 | 0.085 | -0.0648 | No |
| 15 | ADD3 |  |  | 1118 | 0.081 | -0.0713 | No |
| 16 | ACTR2 |  |  | 1224 | 0.075 | -0.0841 | No |
| 17 | ACLY |  |  | 1333 | 0.069 | -0.0977 | No |
| 18 | PGM1 |  |  | 1389 | 0.067 | -0.1029 | No |
| 19 | QDPR |  |  | 1398 | 0.066 | -0.1004 | No |
| 20 | YKT6 |  |  | 1491 | 0.062 | -0.1118 | No |
| 21 | ATP6V1D |  |  | 1507 | 0.061 | -0.1107 | No |
| 22 | PFKL |  |  | 1526 | 0.061 | -0.1102 | No |
| 23 | TM7SF2 |  |  | 1689 | 0.056 | -0.1334 | No |
| 24 | IDH1 |  |  | 1691 | 0.055 | -0.1304 | No |
| 25 | GPI |  |  | 1849 | 0.051 | -0.1531 | No |
| 26 | VLDLR |  |  | 1952 | 0.048 | -0.1670 | No |
| 27 | SYTL2 |  |  | 2082 | 0.045 | -0.1854 | No |
| 28 | TCEA1 |  |  | 2132 | 0.043 | -0.1910 | No |
| 29 | STC1 |  |  | 2141 | 0.043 | -0.1898 | No |
| 30 | PSAT1 |  |  | 2153 | 0.043 | -0.1891 | No |
| 31 | PSMC2 |  |  | 2255 | 0.040 | -0.2033 | No |
| 32 | PIK3R3 |  |  | 2273 | 0.040 | -0.2038 | No |
| 33 | LTA4H |  |  | 2421 | 0.037 | -0.2257 | No |
| 34 | ATP5MC1 |  |  | 2610 | 0.033 | -0.2545 | No |
| 35 | SERP1 |  |  | 2633 | 0.032 | -0.2562 | No |
| 36 | HMGCS1 |  |  | 2674 | 0.031 | -0.2610 | No |
| 37 | IDI1 |  |  | 2749 | 0.030 | -0.2713 | No |
| 38 | PPIA |  |  | 2777 | 0.029 | -0.2741 | No |
| 39 | PGK1 |  |  | 3012 | 0.025 | -0.3109 | No |
| 40 | NAMPT |  |  | 3040 | 0.024 | -0.3139 | No |
| 41 | UBE2D3 |  |  | 3057 | 0.024 | -0.3151 | No |
| 42 | UFM1 |  |  | 3159 | 0.022 | -0.3303 | No |
| 43 | PDAP1 |  |  | 3316 | 0.018 | -0.3548 | No |
| 44 | GCLC |  |  | 3344 | 0.017 | -0.3582 | No |
| 45 | TPI1 |  |  | 3442 | 0.014 | -0.3732 | No |
| 46 | PSPH |  |  | 3455 | 0.014 | -0.3744 | No |
| 47 | PPP1R15A |  |  | 3474 | 0.013 | -0.3766 | No |
| 48 | ACACA |  |  | 3525 | 0.011 | -0.3841 | No |
| 49 | G6PD |  |  | 3603 | 0.009 | -0.3962 | No |
| 50 | HSPA4 |  |  | 3794 | 0.001 | -0.4271 | No |
| 51 | PSMB5 |  |  | 3869 | -0.002 | -0.4391 | No |
| 52 | ARPC5L |  |  | 3870 | -0.002 | -0.4389 | No |
| 53 | COPS5 |  |  | 3885 | -0.003 | -0.4410 | No |
| 54 | SLC2A3 |  |  | 4169 | -0.020 | -0.4861 | No |
| 55 | PSMC4 |  |  | 4249 | -0.025 | -0.4975 | No |
| 56 | EIF2S2 |  |  | 4252 | -0.025 | -0.4964 | No |
| 57 | HSPA9 |  |  | 4311 | -0.030 | -0.5042 | No |
| 58 | ATP2A2 |  |  | 4371 | -0.034 | -0.5119 | No |
| 59 | SSR1 |  |  | 4490 | -0.044 | -0.5286 | No |
| 60 | TFRC |  |  | 4532 | -0.047 | -0.5326 | No |
| 61 | ACTR3 |  |  | 4548 | -0.048 | -0.5323 | No |
| 62 | SLC6A6 |  |  | 4652 | -0.056 | -0.5459 | No |
| 63 | PSMD13 |  |  | 4654 | -0.056 | -0.5429 | No |
| 64 | IFRD1 |  |  | 4671 | -0.057 | -0.5422 | No |
| 65 | SCD |  |  | 4717 | -0.061 | -0.5460 | No |
| 66 | HSPE1 |  |  | 4744 | -0.063 | -0.5467 | No |
| 67 | P4HA1 |  |  | 4761 | -0.065 | -0.5455 | No |
| 68 | PLOD2 |  |  | 4764 | -0.065 | -0.5421 | No |
| 69 | PSMA3 |  |  | 4817 | -0.069 | -0.5466 | No |
| 70 | ERO1A |  |  | 4850 | -0.072 | -0.5477 | No |
| 71 | ENO1 |  |  | 4868 | -0.073 | -0.5463 | No |
| 72 | PSMC6 |  |  | 4872 | -0.073 | -0.5426 | No |
| 73 | PSMA4 |  |  | 4981 | -0.081 | -0.5555 | No |
| 74 | PPA1 |  |  | 5017 | -0.084 | -0.5564 | Yes |
| 75 | IMMT |  |  | 5023 | -0.084 | -0.5524 | Yes |
| 76 | NUP205 |  |  | 5053 | -0.087 | -0.5521 | Yes |
| 77 | CYB5B |  |  | 5098 | -0.092 | -0.5540 | Yes |
| 78 | SERPINH1 |  |  | 5124 | -0.094 | -0.5527 | Yes |
| 79 | UNG |  |  | 5148 | -0.096 | -0.5509 | Yes |
| 80 | CDC25A |  |  | 5172 | -0.099 | -0.5490 | Yes |
| 81 | CYP51A1 |  |  | 5190 | -0.101 | -0.5460 | Yes |
| 82 | PSMD12 |  |  | 5205 | -0.102 | -0.5424 | Yes |
| 83 | UCHL5 |  |  | 5220 | -0.103 | -0.5388 | Yes |
| 84 | ELOVL5 |  |  | 5226 | -0.103 | -0.5336 | Yes |
| 85 | MTHFD2 |  |  | 5256 | -0.107 | -0.5322 | Yes |
| 86 | FADS1 |  |  | 5324 | -0.111 | -0.5368 | Yes |
| 87 | EEF1E1 |  |  | 5432 | -0.122 | -0.5472 | Yes |
| 88 | M6PR |  |  | 5433 | -0.122 | -0.5402 | Yes |
| 89 | XBP1 |  |  | 5441 | -0.123 | -0.5343 | Yes |
| 90 | STIP1 |  |  | 5458 | -0.126 | -0.5296 | Yes |
| 91 | PSMD14 |  |  | 5460 | -0.126 | -0.5226 | Yes |
| 92 | SLC7A5 |  |  | 5488 | -0.131 | -0.5195 | Yes |
| 93 | PSMG1 |  |  | 5518 | -0.133 | -0.5165 | Yes |
| 94 | FADS2 |  |  | 5544 | -0.135 | -0.5128 | Yes |
| 95 | HPRT1 |  |  | 5554 | -0.136 | -0.5064 | Yes |
| 96 | CCNF |  |  | 5562 | -0.137 | -0.4997 | Yes |
| 97 | SLC7A11 |  |  | 5582 | -0.141 | -0.4947 | Yes |
| 98 | GMPS |  |  | 5613 | -0.146 | -0.4912 | Yes |
| 99 | ELOVL6 |  |  | 5663 | -0.154 | -0.4904 | Yes |
| 100 | RPN1 |  |  | 5678 | -0.156 | -0.4837 | Yes |
| 101 | TUBG1 |  |  | 5690 | -0.158 | -0.4764 | Yes |
| 102 | GLA |  |  | 5694 | -0.159 | -0.4677 | Yes |
| 103 | SDF2L1 |  |  | 5697 | -0.160 | -0.4588 | Yes |
| 104 | CACYBP |  |  | 5713 | -0.162 | -0.4520 | Yes |
| 105 | CANX |  |  | 5755 | -0.173 | -0.4487 | Yes |
| 106 | RDH11 |  |  | 5772 | -0.178 | -0.4411 | Yes |
| 107 | TXNRD1 |  |  | 5782 | -0.179 | -0.4322 | Yes |
| 108 | MCM2 |  |  | 5785 | -0.180 | -0.4222 | Yes |
| 109 | PLK1 |  |  | 5836 | -0.192 | -0.4193 | Yes |
| 110 | LDLR |  |  | 5843 | -0.194 | -0.4092 | Yes |
| 111 | HMGCR |  |  | 5906 | -0.208 | -0.4073 | Yes |
| 112 | CCT6A |  |  | 5917 | -0.211 | -0.3968 | Yes |
| 113 | SEC11A |  |  | 5933 | -0.216 | -0.3868 | Yes |
| 114 | FKBP2 |  |  | 5936 | -0.217 | -0.3746 | Yes |
| 115 | DHCR24 |  |  | 5943 | -0.219 | -0.3631 | Yes |
| 116 | TOMM40 |  |  | 5950 | -0.221 | -0.3513 | Yes |
| 117 | HSPD1 |  |  | 5967 | -0.227 | -0.3409 | Yes |
| 118 | SLC2A1 |  |  | 5995 | -0.237 | -0.3317 | Yes |
| 119 | CXCR4 |  |  | 6012 | -0.242 | -0.3204 | Yes |
| 120 | AURKA |  |  | 6026 | -0.248 | -0.3082 | Yes |
| 121 | BUB1 |  |  | 6045 | -0.266 | -0.2958 | Yes |
| 122 | LDHA |  |  | 6057 | -0.272 | -0.2820 | Yes |
| 123 | HSPA5 |  |  | 6061 | -0.274 | -0.2667 | Yes |
| 124 | ACSL3 |  |  | 6078 | -0.284 | -0.2530 | Yes |
| 125 | DHCR7 |  |  | 6083 | -0.287 | -0.2371 | Yes |
| 126 | SQLE |  |  | 6123 | -0.316 | -0.2253 | Yes |
| 127 | PHGDH |  |  | 6126 | -0.318 | -0.2073 | Yes |
| 128 | TMEM97 |  |  | 6131 | -0.324 | -0.1894 | Yes |
| 129 | EBP |  |  | 6133 | -0.324 | -0.1709 | Yes |
| 130 | DDX39A |  |  | 6137 | -0.328 | -0.1526 | Yes |
| 131 | CALR |  |  | 6153 | -0.344 | -0.1352 | Yes |
| 132 | MCM4 |  |  | 6171 | -0.372 | -0.1166 | Yes |
| 133 | CD9 |  |  | 6176 | -0.378 | -0.0955 | Yes |
| 134 | RRM2 |  |  | 6199 | -0.438 | -0.0739 | Yes |
| 135 | DHFR |  |  | 6200 | -0.441 | -0.0486 | Yes |
| 136 | CTSC |  |  | 6224 | -0.509 | -0.0231 | Yes |
| 137 | HSP90B1 |  |  | 6225 | -0.509 | 0.0062 | Yes |
Table: GSEA details [plain text format]

  

Fig 2: HALLMARK\_MTORC1\_SIGNALING: Random ES distribution      
 Gene set null distribution of ES for **HALLMARK\_MTORC1\_SIGNALING**
